# Genome-Wide Identification and Characterization of Soybean *GmLOR* Gene Family and Expression Analysis in Response to Abiotic Stresses

**DOI:** 10.3390/ijms222212515

**Published:** 2021-11-19

**Authors:** Yisheng Fang, Dong Cao, Hongli Yang, Wei Guo, Wenqi Ouyang, Haifeng Chen, Zhihui Shan, Zhonglu Yang, Shuilian Chen, Xia Li, Limiao Chen, Xinan Zhou

**Affiliations:** 1 Key laboratory of Biology and Genetic Improvement of Oil Crops, Ministry of Agriculture and Rural Affairs, Oil Crops Research Institute of Chinese Academy of Agricultural Sciences, Wuhan 430062, China; fangyisheng1111@163.com (Y.F.); yanghongli@caas.cn (H.Y.); vivi1998@126.com (W.G.); oywqgenetic@163.com (W.O.); 18672959732@163.com (H.C.); danzhihui@caas.cn (Z.S.); yangzhonglu@126.com (Z.Y.); chenshuilian@163.com (S.C.); caodong@caas.cn (D.C.); 2 National Key Laboratory of Crop Genetic Improvement, Hubei Hongshan Laboratory, College of Plant Science and Technology, Huazhong Agricultural University, No. 1 Shizishan Road, Hongshan District, Wuhan 430070, China; xli@mail.hzau.edu.cn

**Keywords:** soybean, LOR gene family, phylogenetic analysis, gene expression, osmotic stress, salt stress

## Abstract

The LOR (*LURP*-one related) family genes encode proteins containing a conserved LOR domain. Several members of the LOR family genes are required for defense against *Hyaloperonospora parasitica* (*Hpa*) in Arabidopsis. However, there are few reports of LOR genes in response to abiotic stresses in plants. In this study, a genome-wide survey and expression levels in response to abiotic stresses of 36 LOR genes from *Glycine max* were conducted. The results indicated that the *GmLOR* gene family was divided into eight subgroups, distributed on 14 chromosomes. A majority of members contained three extremely conservative motifs. There were four pairs of tandem duplicated *GmLORs* and nineteen pairs of segmental duplicated genes identified, which led to the expansion of the number of *GmLOR* genes. The expansion patterns of the *GmLOR* family were mainly segmental duplication. A heatmap of soybean LOR family genes showed that 36 *GmLOR* genes exhibited various expression patterns in different tissues. The cis-acting elements in promoter regions of *GmLORs* include abiotic stress-responsive elements, such as dehydration-responsive elements and drought-inducible elements. Real-time quantitative PCR was used to detect the expression level of *GmLOR* genes, and most of them were expressed in the leaf or root except that *GmLOR6* was induced by osmotic and salt stresses. Moreover, *GmLOR4/10/14/19* were significantly upregulated after PEG and salt treatments, indicating important roles in the improvement of plant tolerance to abiotic stress. Overall, our study provides a foundation for future investigations of *GmLOR* gene functions in soybean.

## 1. Introduction

The growth and productivity of plants are affected by adversely environmental factors, including drought, salinity, and extreme temperature [1,2,3,4,5]. Among abiotic stresses, drought and salinity are majorly adverse environmental stresses that limit crop quality and yield in agriculture and threaten food security [6].

The LOR (*LURP*-one related) gene family contains a pfam 04525 domain (DUF567) defined by the Pfam database [7]. It is found in plants, fungi, eubacteria, and some archaea [8]. Previously, the LOR family had fifteen members in *Arabidopsis thaliana* [9]. *AtLURP1* (*LATE UP-REGULATION IN RESPONSE TO HYALOPERONOSPORA PARASITICA 1*) belongs to the LOR gene family. The expression level of *AtLURP1* is very low under normal conditions. However, it is significantly induced more than 30-fold 48 h after *Hyaloperonospora Parasitica* (*Hpa*) infection. As a salicylic acid (SA) marker gene, *AtLURP1* is required for basal defense against *Hpa* and resistance to this pathogen mediated by the R-proteins RPP4 and RPP5 by an SA-dependent pathway [10,11]. Besides, *AtLURP1*, as a target gene of *AtNAC4*, acts as negative regulators of cell death in response to pathogen infection [12]. *AtLOR1* showed a strongly constitutive expression and a highly significant role in basal defense against Hpa [9]. The protein structure of AtLOR1 had been solved by X-ray crystallography (PDB-code:1zxu), which provided a representative structure model for this family. The structure comprised a 12-stranded beta barrel with a central C-terminal alpha helix. The structure model showed that the sequence conservation lies within the secondary structures, as well as the β-hairpin turns between strands 2–3 and 4–5 [8]. These studies reveal the potential important functions of the LOR family genes in plant response to environmental cues.

Soybean (*Glycine max*) is a legume crop grown worldwide that serves as important sources of protein and oil [13,14]. The genome sequence of Williams 82 (a soybean cultivar) was completed in 2010 [15] and has thus provided an opportunity to identify and characterize the gene family of *GmLORs* in soybean. To date, no data are available about the analyses of the LOR gene family in soybean, and their functions on the abiotic stress response are unknown. In the present study, a genome-wide survey of the soybean genome sequence identified 36 *GmLOR* genes. Subsequently, the phylogenetic relationships, gene structures, conserved motifs, duplication patterns, tissue expression patterns, and cis-elements were investigated, and we further analyzed the *GmLOR* genes in response to osmotic and salt stresses. The systematic analysis of the *GmLOR* gene family provides a basis for the further investigation of their functions in soybean.

## 2. Results

### 2.1. Identification of GmLOR Genes in Soybean

A total of 36 *GmLOR* genes were obtained from the soybean genome (Wm82, a2.v1) in the Phytozome v12.1 database, and all of the putative protein sequences were submitted to The Conserved Domain Database (CDD) (https://www.ncbi.nlm.nih.gov/cdd/ accessed on 12 June 2021) to confirm the conserved LOR domain. Finally, 36 *GmLOR* family genes were identified and named *GmLOR1* to *GmLOR36* according to their chromosomal locations (Table S1). Table S1 shows detailed information of GmLOR genes including the number of introns, transcript number, and protein physicochemical characteristics. In the *GmLOR* gene family, *GmLOR17/18/22/26* contain two transcripts, *GmLOR5/24* contain three transcripts, and the rest of the members contain one transcript. The full length of GmLOR proteins ranged from 109 (GmLOR6) to 314 (GmLOR28) amino acids (aa) with the molecular weight (Mw) varying from 12.4 to 34.8 kDa and the isoelectric point (pI) varying from 3.96 (GmLOR31) to 9.64 (GmLOR4). 

### 2.2. Phylogenetic Relationships of LOR Proteins in Soybean and Arabidopsis

**Figure 1:**
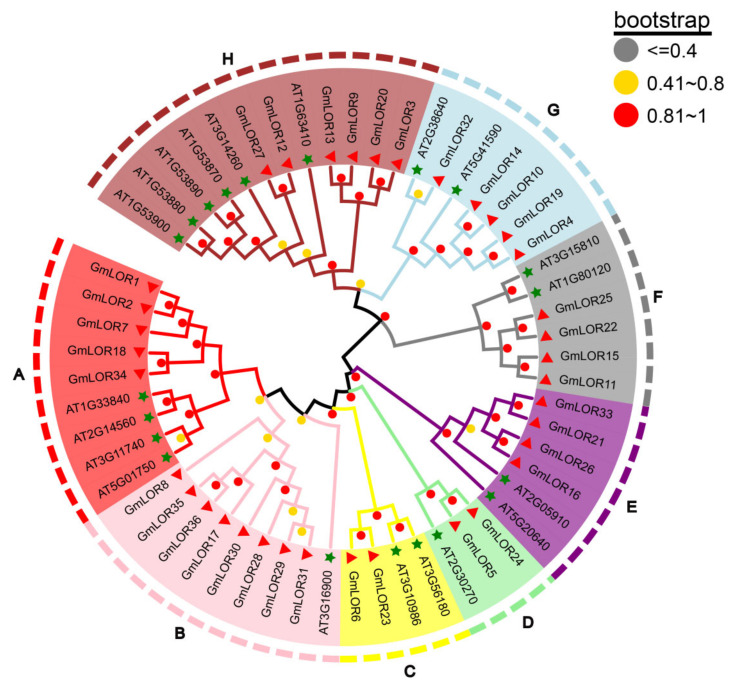
Phylogenetic analysis of soybean and Arabidopsis LOR proteins. The tree was conducted based on the full-length amino acid sequences using MEGA 6.0 by the neighbor-joining method with 1000 bootstrap replicates. Bootstrap values are shown in different-colored solid circles on the branches. Bootstrap values (1000 repetitions) less than 0.41 are represented by gray solid circles, those between 0.41 and 0.8 are represented by yellow solid circles, and those above 0.81 are represented by red solid circles in the corresponding branches. The green five-pointed star represents AtLOR family members. The red triangle represents GmLOR family members. The tree shows eight major phylogenetic subgroups (A to H) indicated with different colored backgrounds.

To determine the evolutionary relationships of LOR protein families in soybean and Arabidopsis, an unrooted neighbor-joining tree was constructed. The 36 GmLORs were classified into 8 distinct subgroups (A–H) together with 20 AtLORs from Arabidopsis (Figure 1). Each subgroup contains 3–12 members. The same subgroup contains soybean LOR proteins and AtLOR proteins, suggesting the possible conservation of function within dicot species. In general, the same subgroup may have similar functions, which helps us to study the potential biological functions of the GmLORs family. In subgroup A, five proteins of the GmLOR family members (GmLOR1/2/7/8/34) clustered with four proteins of the AtLOR family members, including AtLURP1 (At2G14560) and AtLOR1 (At5G01750). *AtLURP1* plays a crucial role in *HPa* pathogen defense and regulates cell death in pathogen infection [10,11,12]. *AtLOR1* revealed a significant role in basal defense against Hpa [9]. The biological functions of other subgroup members have not been reported. There are two AtLOR family members (AT1G53880 and AT1G53900) in subgroup H that contain the eukaryotic translation initiation factor 2B (eIF-2B, PF01008) domain besides the LOR domain.

### 2.3. Gene Structures and Protein Motifs of GmLOR Family

**Figure 2:**
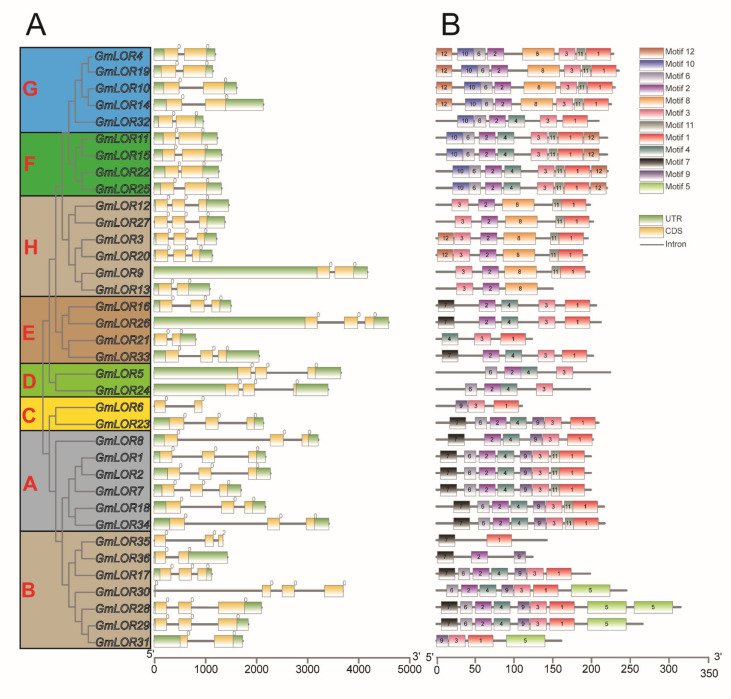
Protein motifs and gene structures of the soybean LOR family. According to the phylogenetic relationships, the GmLOR genes clustered into eight major phylogenetic subgroups (**A**). The exon/intron structures of GmLOR genes. The relative position and size of the exon can be estimated using the scale at the bottom. Yellow boxes, gray lines, and green boxes represent exons, introns, and UTR, respectively (**B**). The putative conserved motifs in GmLORs. Different motifs and their relative positions are represented by the colored boxes.

By screening the sequences and annotation files, gene structures of the *GmLORs* family were obtained (Figure 2A). The numbers of introns and exons in *GmLOR* genes ranged from 1 to 3 and from 2 to 4, respectively. Among the *GmLOR* genes, more than half of the genes had two introns, 15 out of 36 genes had a single intron, and only one gene (*GmLOR30*) had three introns.

A total of 12 conserved motifs among the GmLOR proteins were identified using MEME suite (Figure 2B). Motifs 1 to 12 were designated based on the E-value of each motif. The details of 12 putative conserved motifs are displayed in Figure S1. Among them, motifs 1, 2, and 3 are the three most conserved motifs and are presented in almost all subgroups in the GmLOR family. Among them, common motif 1 and motif 2 were located in the C-terminal and N-terminal of the GmLOR protein family, respectively, which was found in 32 out of 36 (89%) GmLOR proteins. Motif 3 was shown in 34 out of 36 (94%) GmLORs. Motif 3 is closer to the C-terminus except for GmLOR31 and all members of the H subgroups, which are closer to the N-terminus. The results indicated that these three conserved motifs are essential in the GmLOR family. Some motifs were only found in specific subgroups. For example, motif 5 was only found in subgroup B, and motif 8 was found in subgroups G and H. Motif 10 was found in subgroups F and G. Motif 12 was found in subgroups F, G, and H. The above results suggested that functional divergence existed in different soybean LOR subgroups.

### 2.4. Chromosomal Distribution and Expansion Patterns of Soybean LOR Genes

**Figure 3:**
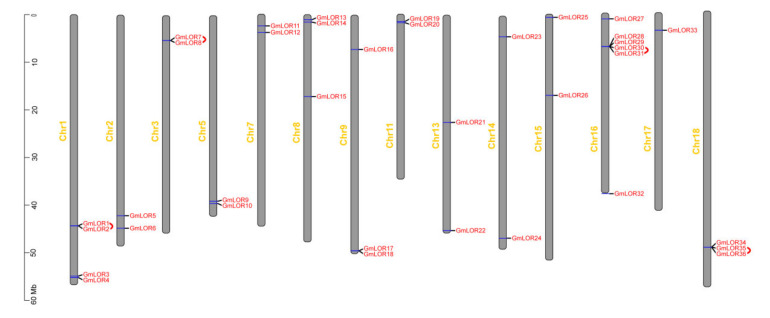
Chromosomal distribution and tandem duplications of GmLORs. The 36 GmLORs were mapped onto soybean chromosomes based on their physical positions. Four tandemly duplicated gene-pairs are labeled by red lines. The scale on the left is in megabases (Mb).

TBtools software was used to illustrate the physical positions of *GmLOR* genes on soybean chromosomes. The results showed that 36 *GmLOR* genes were unevenly distributed on 14 chromosomes (Figure 3). Among 20 chromosomes, Chromosome 16 contained the most *GmLOR* genes, and six genes were located on this chromosome. Chromosome 17 contained a single *GmLOR* gene. The other chromosomes possessed two to four *GmLOR* genes. Chromosomes 4, 6, 10, 12, 19, and 20 carried no *GmLOR* genes. A quantity of 33 out of 36 (90%) *GmLOR* genes were located on the chromosome arms except for *GmLOR15/21/26*. The result is in agreement with the genome-wide gene distribution pattern, where approximately 78% of all soybean genes are located on the chromosome arms [15].

**Figure 4:**
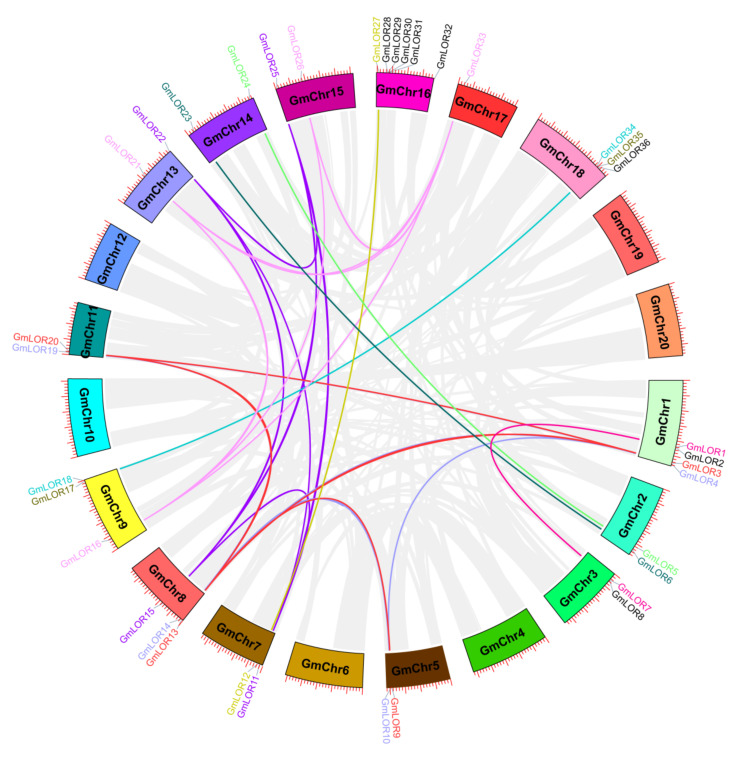
Distribution and synteny analysis of GmLOR genes. The soybean chromosomes are represented in different-colored partial circles. The different colored bars instruct LOR syntenic regions on the GmLOR gene family. The gray line represents other gene syntenic regions in the soybean genome.

In the expansion of the genome, gene duplication events are considered as the major causes that contributed to the expansion of gene families [16]. Soybean is a diploidized ancient tetraploid, which underwent two whole genome duplications [17]. Most soybean genes are paralogous genes with multiple copies. Thus, we analyzed the duplication events of Whole Genome Duplication (WGD)/segmental duplication and tandem duplication in *GmLOR* genes (Figure 3 and Figure 4) to obtain more information about the expansion of *GmLOR* genes in the soybean genome. The results revealed that 23 out of 36 (64%) *GmLOR* genes expanded through segmental duplication. Four pairs (*GmLOR1/2*, *GmLOR7/8*, *GmLOR30/31*, and *GmLOR35/36*) expanded through tandem duplication. *GmLOR1/7* and *GmLOR17/35* were also expanded through WGD/segmental duplication. Most of the *GmLOR* genes have more than one pair of duplication events, for example, *GmLOR4* and *GmLOR11* have three duplicated genes (Figure 4, Table S2). Together, these results indicated that WGD/segmental duplication is the main driving force for the large expansion of *GmLOR* genes in the soybean genome.

The expansion time of GmLOR family genes were further estimated according to the pairwise synonymous distances (Table S2). Based on a previous study, Ks values of 0.06–0.39 correspond to the 13 Ma-ago (Mya) glycine-lineage-specific genome duplication, Ks values of 0.40–0.80 correspond to the 59 Mya early legume WGD, and Ks values greater than 1.5 probably correspond to the more ancient “gamma” event. These results showed that 11 duplicated *GmLOR* pairs (with Ks values of 0.07–0.32) were associated with the 13 Mya glycine-lineage-specific genome duplication. A quantity of 10 *GmLOR* pairs (with Ks values of 0.41–0.74) were associated with the 59 Mya early legume duplication. Otherwise, *GmLOR1/GmLOR2* and *GmLOR30/GmLOR31* were associated with less than 13 Mya based on the Ks value. In addition, *GmLOR* orthologous gene pairs displayed Ka/Ks < 1 except *GmLOR30/GmLOR31* (Table S2). Therefore, we speculated that the majority of the *GmLOR* gene family might go through purifying selective pressures during the evolution.

### 2.5. Cis-Elements Analysis of the GmLOR Promoters

**Figure 5:**
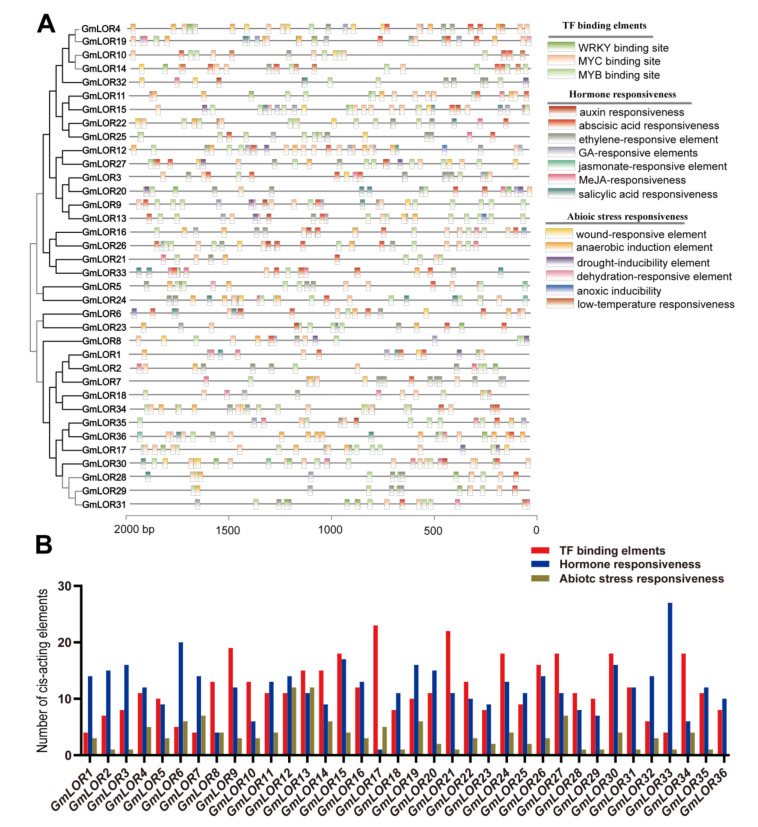
The analysis of the cis-elements in the promoter regions of GmLOR genes. (**A**) Predicted cis-elements in the 2000 bp upstream regions of GmLOR genes and three main categories of cis-elements. The names of the cis-elements are indicated by different-colored boxes. The scale indicates the relative position of each cis-element relative to the start codon, ATG. (**B**) Three categories of cis-elements presented in the promoter regions of GmLOR genes.

Cis-acting elements in the promoter region might play a key role in response to plant growth and environmental stresses by transcriptional regulation of gene expression [18]. In this research, the 2000 bp upstream sequences of GmLOR genes were extracted via the PlantCARE database (Figure 5A and Table S3). Apart from the abundant amount of core promoter (TATA-box) and enhancer elements (CAAT-box), four types of cis-elements were identified, such as hormone response, transcription factors (TFs) binding site, and stress response (Figure 5A). Putative main TF binding elements include MYB, WRKY, and MYC. The plant hormones response elements contain abscisic acid (ABA), salicylic acid (SA), jasmonate (JA), methyl jasmonate (MeJA), gibberellic acid (GA), ethylene, and auxin. The wound-, drought-, dehydration-, cold-, anoxic-, and defense stress-responsive elements were detected in the promoter of GmLOR genes. In addition, three kinds of cis-elements are shown in Figure 5B. The promoter regions of GmLOR17 and GmLOR33 have a maximum number of TF binding elements and hormone response elements, respectively.

### 2.6. Expression Patterns of GmLOR Members in Different Soybean Tissues

**Figure 6:**
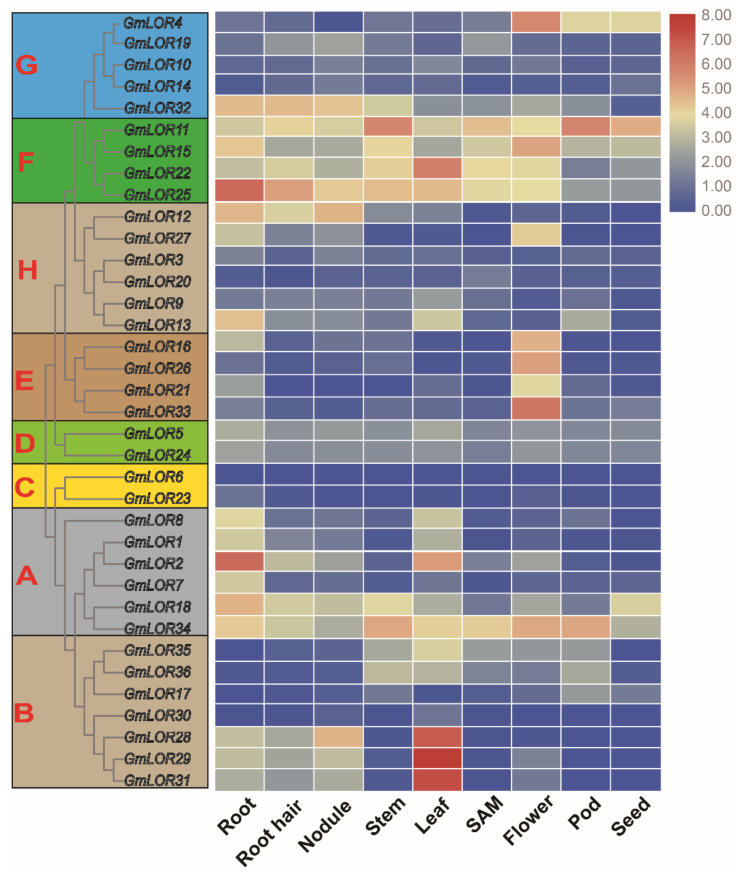
Expression profile of 36 GmLOR genes in different tissues of soybean. FPKM values of GmLOR genes were obtained from Phytozome v12 and were transformed by log2. Different colors in the heat map represent gene transcript abundance values, as shown in the bar at the top of figure.

To understand the potential functions of GmLOR family members, the RNA-seq data (FPKM values) of GmLORs from Phytozome V12.1 are useful for investigating the expression pattern of GmLOR genes in different soybean tissues, including shoot apical meristem (SAM), root, root hair, stem, leaf, flower, pod, nodule, and seed (Table S4). A heatmap of 36 *GmLOR* gene expressions in nine tissues was constructed by EvolView (Figure 6). The results showed that all 36 *GmLOR* genes except *GmLOR6* were expressed in one tissue at least, and *GmLOR34* and all of the subfamily F members (*GmLOR11/15/22/25*) exhibited high expression in multiple tissues. However, the expression levels of some genes, such as *GmLOR3/9/10/14/17/19/20/23/30*, were very low in all of nine tissues. In addition, some *GmLOR* genes, such as *GmLOR27,* showed tissue-specific expression under normal conditions, and all of the subgroup E members (*GmLOR16/21/26/33*) exhibited a higher expression level in the flower than other tissues. *GmLOR28/29/31/35/36* were highly expressed in leaves, and *GmLOR12* was highly expressed in the root system, including the root, root hair, and nodule.

### 2.7. Transcriptional Responses of GmLORs in Response to Abiotic Stresses

**Figure 7:**
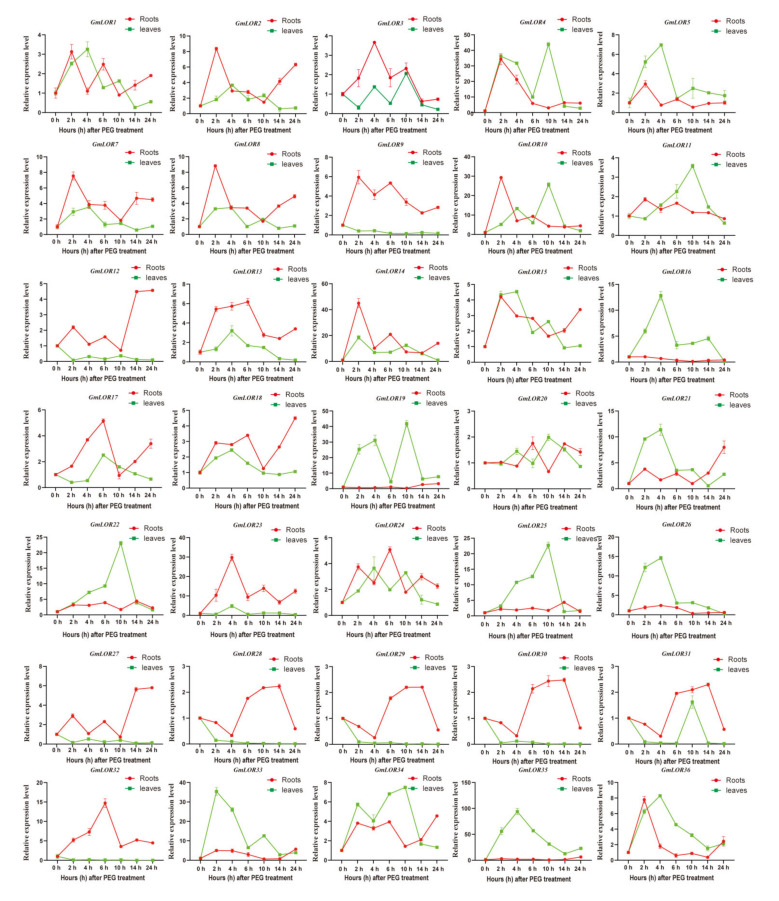
Relative expression levels of GmLORs in response to PEG stress. The soybean cultivar, Tianlong 1, was grown hydroponically, cultured for two weeks, and then exposed to 25% PEG treatment. The relative expression levels of 36 GmLOR genes at 2, 4, 6, 10, 14, and 24 h were determined by qRT-PCR in comparison with 0 h (the expression was set as “1”). The soybean ACT11 gene was used as the internal control. The data represented the mean ± SD of three independent biological repetitions.

As some abiotic stress-responsive elements were detected in the promoters of *GmLOR*s (Figure 5), we speculated that these genes may mediate the plant response to abiotic stresses. To validate the involvement of *GmLOR*s in response to osmotic or salt stress, the expressions of *GmLOR* genes at 0 h, 2 h, 4 h, 6 h, 10 h, 14 h, and 24 h after PEG or salt treatments were detected. The results showed that most *GmLOR* genes exhibited various expression levels under PEG treatment (Figure 7). The expressions of *GmLOR4/16/19/21/26/33/35* and *GmLOR4/10/14/23* were greatly upregulated above 10 times by PEG in the leaf and root at the early stage. For example, *GmLOR4/10/14/33* expression rapidly elevated and reached a peak at 2 h after PEG treatment. In contrast, *GmLOR4/10/19/22/25/34* expression levels were gradually increased and were induced above 10 times in the leaf at the late stage. Notably, the expression of *GmLOR16/19/35* changed specifically in the leaf and that of *GmLOR9/32* changed specifically in the root in response to PEG treatment.

**Figure 8:**
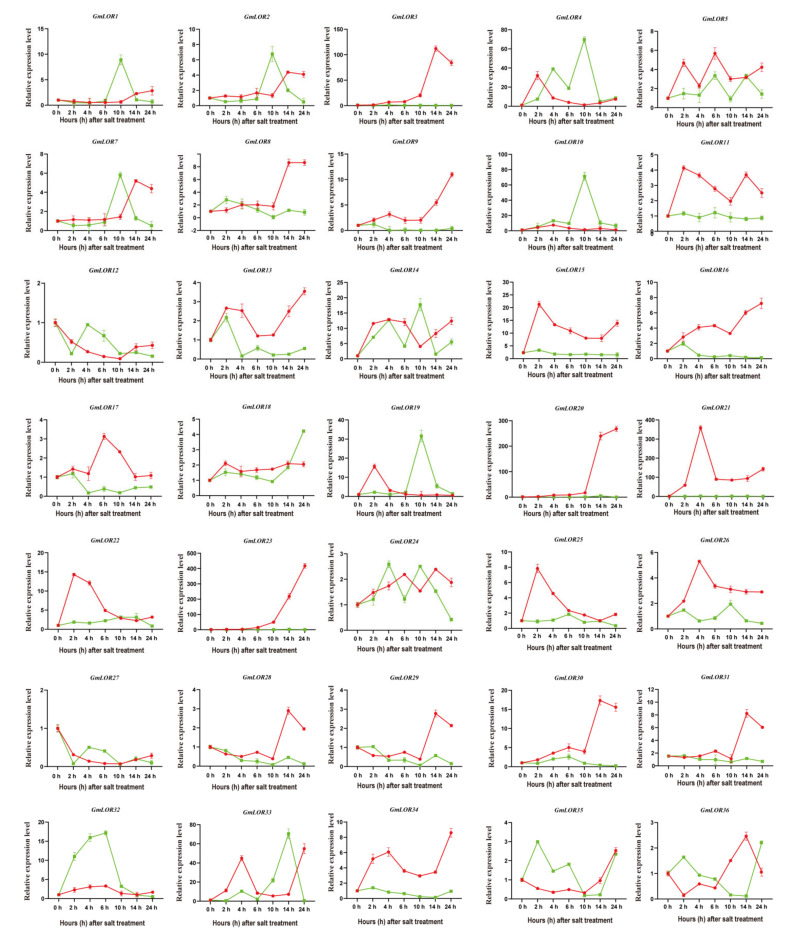
Relative expression levels of GmLORs in response to salt stress. The relative expression levels of 36 GmLOR genes at 2, 4, 6, 10, 14, and 24 h were determined by qRT-PCR in comparison with 0 h (the expression was set as “1”). The soybean ACT11 gene was used as the internal control. The data represent the mean ± SD of three independent biological repetitions.

The qRT-PCR was also performed to analyze relative expressions of *GmLOR* family members under 200 mM of NaCl treatment. Compared with *GmLOR* genes in response to PEG treatment, the majority of *GmLORs* were significantly expressed in the root (Figure 8), which senses salt stress signals. Some induced genes (*GmLOR3/8/9/16/20/23/28/29/30/31*) reached the maximum at the later stages, which is different from the results under PEG treatment. The expressions of *GmLOR3/20/21/23* were induced only in the roots, not the leaves. *GmLOR12/28/31* showed reduced expression in the leaves, while *GmLOR17/27/29* displayed reduced expression in both the roots and leaves.

Among these *GmLORs*, *GmLOR6* showed no expression in the root and leaves under nonstress and stress treatments, which is consistent with the FPKM data from the Phytozome database. Based on those results, most *GmLOR* members may be involved in response to PEG or salt stresses in soybean. Moreover, the induction of *GmLOR4/10/14/19/33* by abiotic stresses in the roots and leaves indicates that they might play potential roles in the response to abiotic stresses of soybean.

## 3. Discussion

With the availabilities of the whole genome sequence of many plants, the majority of gene families have been identified [19]. However, little is known about the LOR family. In the current study, we identified the LOR family in soybean, including phylogenetic relations, gene structures, putative conserved motifs, chromosomal locations, gene duplications, synteny analyses, and cis-elements in *GmLOR* promoters. Besides, the expression patterns of the *GmLOR* genes in various tissues under nonstress conditions and in response to abiotic stresses were explored. Our study provides a foundation for future investigation of GmLOR gene functions in soybean. 

The LOR gene family, which is present in plants, fungi, eubacteria, and some archaea, contains a conserved LOR domain. In our study, all of the GmLOR and AtLOR genes were classified into A–H subgroups (Figure 1). The LOR protein structures of soybean and Arabidopsis were more similar on the same subgroup or branch. We also identified syntenic relationships of *GmLORs* and *AtLORs* family members (Table S5). The results showed that 8 out of 20 *AtLOR* genes and 16 out of 36 *GmLOR* genes were orthologous. Among them, one pair of syntenic orthologous genes (one to one) was identified (*AT2G30270*-*GmLOR24*). Besides, there were also syntenic orthologous gene pairs with one *AtLOR* family gene corresponding to multiple *GmLOR* family genes: *AT1G53870*- *GmLOR12/27*, *AT1G63410*-*GmLOR3/9*, and *AT5G41590*-*GmLOR4/10/14/19*. There were also gene pairs with two *AtLOR* family genes corresponding to the same *GmLOR* family genes: *AT1G80120/AT3G15810-GmLOR11/15/22/25* and *AT2G05910/AT5G20640-GmLOR21/26/33*. The result indicated that the genes were derived from the earlier ancestor of Arabidopsis and soybean. Synteny events of the LOR family suggested that many LOR genes arose before the divergence of the Arabidopsis and soybean lineages.

In previous studies, *AtLURP1* and *AtLOR1* play essential roles in the defense response to *Hpa*, which were located in the same branch. *GmLOR1/2/7/18/34* were located in the same subgroup with *AtLURP1* and *AtLOR1*. It predicted that soybean members in subgroup A may be correlated to plant defense. In general, positive correlations between species genome size and the number of gene family members existed [20,21]. Consistently, GmLOR and AtLOR genes were identified, respectively (Figure 1). Gene structure is differentially adaptive in the evolution of multi-gene families [22]. In this study, we identified the number of introns for 36 *GmLOR* members; 15 members had one intron, 20 members had two introns, and only one gene had three introns (Figure 2A and Table S1). Introns were considered to be a necessary way to acquire new gene functions and preferred to rise at the earlier stages of gene expansion and gradually diminish over time [23,24,25,26]. In previous studies, genes were rapidly activated by a compact gene structure with few introns to respond to various stresses timely [27,28]. Here, most of the *GmLOR* genes were rapidly and highly induced under osmotic and salt stress (Figure 7 and Figure 8), which can approve the standpoints. Alternative splicing (AS) can form more than one mRNA isoform and may change protein activity [29,30]. The transcript numbers of 36 *GmLOR* genes were scanned. Most of them have only one transcript profile; however, *GmLOR17/18/22/26* have two transcripts and *GmLOR5/24* have three transcript profiles. These results suggested that they may play different functions in soybean response to various conditions. A total of 12 putative conserved motifs were identified by MEME online tools analysis (Figure 2B). Motifs 1, 2, and 3 were highly conserved and existed in most GmLORs. The other motifs have a clear preference in some subgroups or clades. For example, motif 5 was unique to GmLOR28/29/30/31. Motif 9 only existed in subgroups A, B, and C; Motif 10 only existed in subgroups F and G. As we know little about the GmLOR family, these putative motifs may also function in the interaction between LOR proteins and their substrates. It is worth investigating the roles of those putative motifs. Motifs 1, 2, and 3 could be important factors that determine the essential molecular functions among the GmLOR family members, and the preference of motifs may be the structural basis for the diversity in gene functions. In short, similar intron numbers and motif arrangements were found in the same subgroups. This correlation of gene structure further confirmed the classifications of the GmLOR gene family.

Ancient duplication events and a high rate of retention of extant pairs of duplicates have contributed to an abundance of duplicate genes in plant genomes [31]. Gene duplication is a major driving force for the expansion of gene families and the evolution of novel functions, such as adaptation to stress and induction of disease [16,31,32]. The presence of two or more genes on the same chromosome indicate a tandem duplication event, while two or more genes present on different chromosomes reveal a segmental duplication event. Tandem duplication and segmental duplications have been considered as the main duplication patterns for gene family expansion [33]. However, segmental duplications occur more frequently because most plants retain abundance duplicated chromosomal blocks within their genomes through polyploidy followed by chromosome rearrangements [34]. In this study, 23 out of 36 *GmLOR* genes underwent segmental duplication. In prior studies, the soybean genome underwent two rounds of segmental duplication in its evolutionary history at approximately 13 and 59 Mya ago, resulting in a highly duplicated genome with nearly 75% of the genes present in multiple copies [15]. Here, 10 duplicated *GmLOR* pairs were associated with 59 Mya early legume duplication. In addition, 11 *GmLOR* pairs were associated with 13 Mya glycine-lineage-specific genome duplication (Table S2). The Ka/Ks ratio is a measure used to examine the mechanisms of gene duplication evolution after divergence from their ancestors [35]. The value gives an insight into the selection pressure on amino acid substitutions, with a Ka/Ks ratio < 1 indicating a purifying selection, a Ka/Ks value of 1 suggesting a neutral selection, and a Ka/Ks value of >1 suggesting a positive selection. Almost all duplication events show Ka/Ks ratio < 1, indicating that the majority of *GmLOR* genes underwent a purifying selection, and only a pair (*GmLOR30/GmLOR31*) might be evolved by positive selection.

Expression pattern analyses could provide insight into the potential functions of genes. Here, RNA-seq data (FPKM values) from the Phytozome database were used to study the expression profiles of *GmLORs* in different tissues from the Phytozome database (Figure 6). On the whole, all of subgroup F members showed highly expressed abundances in nine tissues. Some *GmLOR* subgroups or clades exhibited tissue-specific expression. For instance, the members from subgroup E showed a higher expression level in the flower, subgroup B showed a high expression in the leaves except *GmLOR17/30*. It is worth mentioning that most members showed weak expression abundance in nine tissues under normal conditions; yet, they were notably induced by abiotic stress. Some *GmLOR* genes were ubiquitously expressed in different soybean tissues, implying their essential function. Some *GmLOR* genes may function in particular tissues and may be involved in the life cycle of soybeans. It is well-known that the gene duplication event is a main source of functional differentiation, which greatly increases functional diversity and improves the adaptability of species to the environment [36]. Here, we compared the expression abundances of the duplicate genes in different tissues. Among the 23 pairs of duplicate genes, 70% (16/23) of the duplicate gene pairs showed a similar expression pattern, suggesting that they might have functional redundancy. Some of duplicated gene pairs have different expression patterns. For example, *GmLOR2* had higher expression in the roots and leaves than *GmLOR7*. *GmLOR13* had higher expression in the roots, leaves, and pods than its paralogs *GmLOR3/9/20*. *GmLOR10/14/19* had weak expression in all the detected tissues; yet, its paralog *GmLOR4* had higher expression in the flowers, pods, and seeds. These results indicated that strengthened functionalization or neo-functionalization could occur during the duplication process of LOR genes in soybean.

Cis-elements play important roles in the transcriptional regulation of genes involved in response to different environmental stress [37,38]. In a previous study, the key pathogen-response elements are located between positions −85 and −46 of *AtLURP1* promoter. LURP^−85 to −46^ has a w-box (a WRKY binding to site). The expression of *AtLURP1* was regulated by *AtWRKY70* binding w-box [39]. Here, the cis-elements in the promoter of *GmLOR* genes were analyzed, which were classified as three main categories as TF binding site, hormone responsiveness, and stress responsiveness (Figure 5A). It suggested the *GmLOR* family might participate in regulating plant stress responses. The cis-element number statistics of *GmLOR* genes showed that every *GmLOR* gene has a different putative cis-element number, including the duplicated gene pairs (Figure 5B). This result could partly explain the different expression patterns of duplicated genes or different stress responses. Collectively, all kinds of cis-elements distributed widely throughout the promoter regions of *GmLOR* family genes, revealing that *GmLOR* may have intricate expression profiles and play important roles in the regulation of soybean stress resistance.

In Arabidopsis, *AtLURP1* and *AtLOR1* had been reported to participate in biotic stress [9,10,11,12]. However, whether or not there is a LOR family response to abiotic stresses in plants remains unclear. In our study, we identified the transcriptional responses of 36 soybean LOR genes in response to osmotic and salt stresses by qRT-PCR. *GmLOR4/10/14/16/19/21/22/23/2526/32/33/35* genes (>10 folds) were significantly upregulated under PEG treatment. In addition, *GmLOR3/4/9/10/14/15/19/20/21/23/30/32/33* genes (>10 folds) were also significantly upregulated under salt treatment. These results suggest that these genes may play an important role in the response to osmotic and salt stresses. Combined with the qRT-PCR data of osmotic and salt stresses, *GmLOR4/10/14/19* genes located in the same subgroup and belonging to segmental duplicated gene pairs had a significantly positive response to PEG and salt stresses. Together, these genes might play potential roles in response to osmotic and salt stresses and they can be used as important candidate genes to genetically engineer plant fitness for osmotic and salinity conditions.

## 4. Materials and Methods

### 4.1. Identification of the LOR Gene Family Members in Soybean 

The whole LOR protein sequences from soybean were downloaded from Ptytozome v12.1 (http://phytozome.jgi.doe.gov/pz/portal.html, accessed on 12 June 2021). The Hidden Markov Model (HMM) profile of the LOR family domain PF04525 (http://pfam.xfam.org/family/PF04525, accessed on 12 June 2021) was used as a query in a BLASTP (*p* < 0.001) to search predicted LOR proteins of the soybean genome database [40]. All candidate genes were examined to contain the conserved LOR domain (PF04525) using Pfam 32.0 (Protein family: http://pfam.xfam.org/, accessed on 12 June 2021) and CDD (https://www.ncbi.nlm.nih.gov/cdd/, accessed on 12 June 2021) [41]. Similarly, the whole putative AtLOR family members were also confirmed and those protein sequences were used for phylogenetic analysis with the GmLOR family. All of the putative GmLOR amino acid sequences were analyzed by ExPASy (https://web.expasy.org/protparam/, accessed on 12 June 2021) to calculate MW and pI [42].

### 4.2. Sequence Alignment and Phylogenetic Analysis of LOR Proteins

The putative amino acid sequences of GmLOR and AtLOR were used for phylogenetic analysis. Multiple sequence alignments were performed using by Clusstal X (v2.0) with the default parameters. The alignment results were used to construct a phylogenetic tree with MEGA 6.0 [43] based on the neighbor-joining method with the bootstrap test of 1000 times and the pair-wise option. The phylogenetic tree was visualized by Evolview v2 (https://evolgenius.info//evolview-v2/, accessed on 14 June 2021) [44].

### 4.3. Gene Structure and Conserved Motifs Analysis 

The GmLOR exon-intron structure was displayed by the Gene Structure Display Server (GSDS: http://gsds.cbi.pku.edu.cn/index.php, accessed on 14 June 2021) [45]. The conserved motifs of GmLOR protein sequences were predicted by the Multiple Em for Motif Elicitation (MEME: http://meme-suite.org/tools/meme, accessed on 14 June 2021) with the default parameters: zero or one per sequence for distribution of motif occurrences, 12 for the maximum number of motifs, and 6–50 amino acids for optimum motif width [46].

### 4.4. Chromosomal Location and Gene Duplication

The chromosomal positions of *GmLOR* genes were obtained from SoyBase (http://soybase.org/, accessed on 15 June 2021) [47]. TBtools software [48] was used for the mapping of *GmLOR* genes’ chromosomal position and relative distances. The Multiple Collinearity Scan toolkit (MCScanX) [49] was used to identify the duplication events that occurred in *GmLOR* genes in the soybean genome. In brief, BLASTP was performed to identify the intra-species and inter-species parameters settings: alignment significance: E_VALUE (default: 1 × 10^−5^; MATCH_SCORE: final score (default: 50); MATCH_SIZE: number of genes required to call a collinear block (default: 5) and the maximum gaps (default: 25) [49]. *GmLOR* genes falling in the identified collinear blocks were considered as segmental events, while closely adjacent (no more than one gene separating them) homologous LOR genes were considered to represent tandem duplication events, based on the identification standards in MCScanX. The numbers of synonymous (Ks) and nonsynonymous (Ka) substitutions per site for the intra-species and inter-species of the LOR gene pairs from segmental duplication were calculated using the DnaSP program [50]. Finally, the syntenic relationships of LOR genes were illustrated with the CIRCOS program (http://circos.ca/, accessed on 17 June 2021) [51].

### 4.5. Putative Cis-Elements in the Promoter Regions

The 2000 bp sequences upstream from the translation start codon of all of the *GmLOR* genes were obtained from Phytozome v12.1 [52]. The putative stress or hormone-responsive *cis*-acting regulatory elements in these sequences were predicted using the PlantCARE online database (http://bioinformatics.psb.ugent.be/webtools/plantcare/html/, accessed on 18 June 2021) [53].

### 4.6. GmLOR Gene Expression Patterns in Soybean Different Tissues

The expression levels of 36 *GmLOR* genes in nine soybean tissues were obtained from Fragments Per Kilobase per Million (FPKM) values at Phytozome v12.1 [52]. A heatmap of *GmLOR* genes was constructed using the Evolview v2 online database (https://www.evolgenius.info//evolview/, accessed on 20 June 2021), to visualize the expression levels in different tissues based on the log2 (FPKM) values of *GmLOR* genes.

### 4.7. Plant Materials and Abiotic Stress Treatments

Soybean Tianlong 1 seeds sterilized in 1% sodium hypochlorite for 1 min, followed by three washes in sterilized water, were germinated in a growth chamber for 5 days at a 14 h/10 h (light/dark) photoperiod, 25 °C, and 60% relative humidity. After 5 days, uniformly grown seedlings were transferred into holes of foam board on a plastic box containing distilled water and grown until the two soybean leaves fully unfolded. The seedlings at V1 stages were treated with 25% PEG or 200 Mm of sodium chloride solution. The root and true leaf were collected at 0, 2, 4, 6, 10, 14, and 24 h, immediately frozen in liquid nitrogen, and then stored at −80 °C for gene expression analysis. Three biological replicates were obtained from each time point.

### 4.8. Expression Analysis of Soybean GmLOR Genes

Total RNA was extracted from the root and young leaf of soybean under nonstressed, PEG, and salt treatments using TRIzol reagent (Invitrogen, Waltham, MA, USA) according to the manufacturer’s instruction. Approximately 1 μg of total RNA was reverse-transcribed to first-stand cDNA using HiScript II the 1st strand cDNA synthesis kit (Vazyme, Nanjing, China). The specific primers of *GmLOR* genes were designed by primer premier 5.0 (Premier Biosoft International, Palo Alto, CA, USA) software for RT-PCR (Table S6). All primers were synthesized by Sangon Biotech (Shanghai, China). A soybean housekeeping gene *Actin11* was used as the internal reference gene to calculate the relative expression levels of *GmLOR* genes. The qRT-PCR was carried out using the SYBR Premix ExTaqTM kit (Takara, Kusatsu, Japan) on a Bio-Rad CFX96 Real-Time PCR system. Each qRT-PCR reaction was conducted in a 20 μL reaction volume including 10 μL of 2X SYBR Premix Ex Taq, 0.5 μL of a 10 μM solution of each primer, 1 μL of diluted cDNA, and 8 μL of RNAse-free water. The PCR program was set as follows: 95 °C for 5 min; 40 cycles of 95 °C for 10 s, 60 °C for 10 s, and 72 °C for 20 s. Three technical replicates and three biological repeats were performed for each sample. To determine the relative expression levels of *GmLOR* genes in response to abiotic stress, the expression levels of each gene in different tissues under nonstressed conditions were set to “1.” The relative expression levels of *GmLOR* genes in soybean root and young leaf at 2, 4, 6, 10, 14, and 24 h compared with 0 h after PEG or salt treatments were calculated using the 2^−ΔΔCT^ method [54].

## 5. Conclusions

In this work, we performed the first genome-wide identification of the soybean LOR family and described a detailed and multi-level investigation of their phylogenetic relationship, gene structure, motif compositions, chromosomal distribution, expansion patterns, *cis*-acting elements, and tissue expression patterns. We also characterized the expression of *GmLOR* genes in response to osmotic and salt stresses. This study underlies further work to dissect the characteristics of the *GmLOR* gene family and simultaneously accelerates functional research of their roles in soybean stress tolerance.

## Data Availability

The datasets used and/or analyzed during the current study are available from the corresponding author on reasonable request. However, most of the data are shown in Supplementary Materials.

## Supplementary Materials

The following are available online at https://www.mdpi.com/article/10.3390/ijms222212515/s1.
