# Supplementary material for: Genome-Wide Identification and Characterization of Soybean GmLOR Gene Family and Expression Analysis in Response to Abiotic Stresses
Source: Int J Mol Sci. 2021 Nov 19;22(22):12515. doi: 10.3390/ijms222212515 (PMC8624885; doi:10.3390/ijms222212515)
Supplement: Supplementary file 1 [file ijms-22-12515-s001.zip › ijms-1420792-supplementary/Figure S1 and Table S1.pdf]

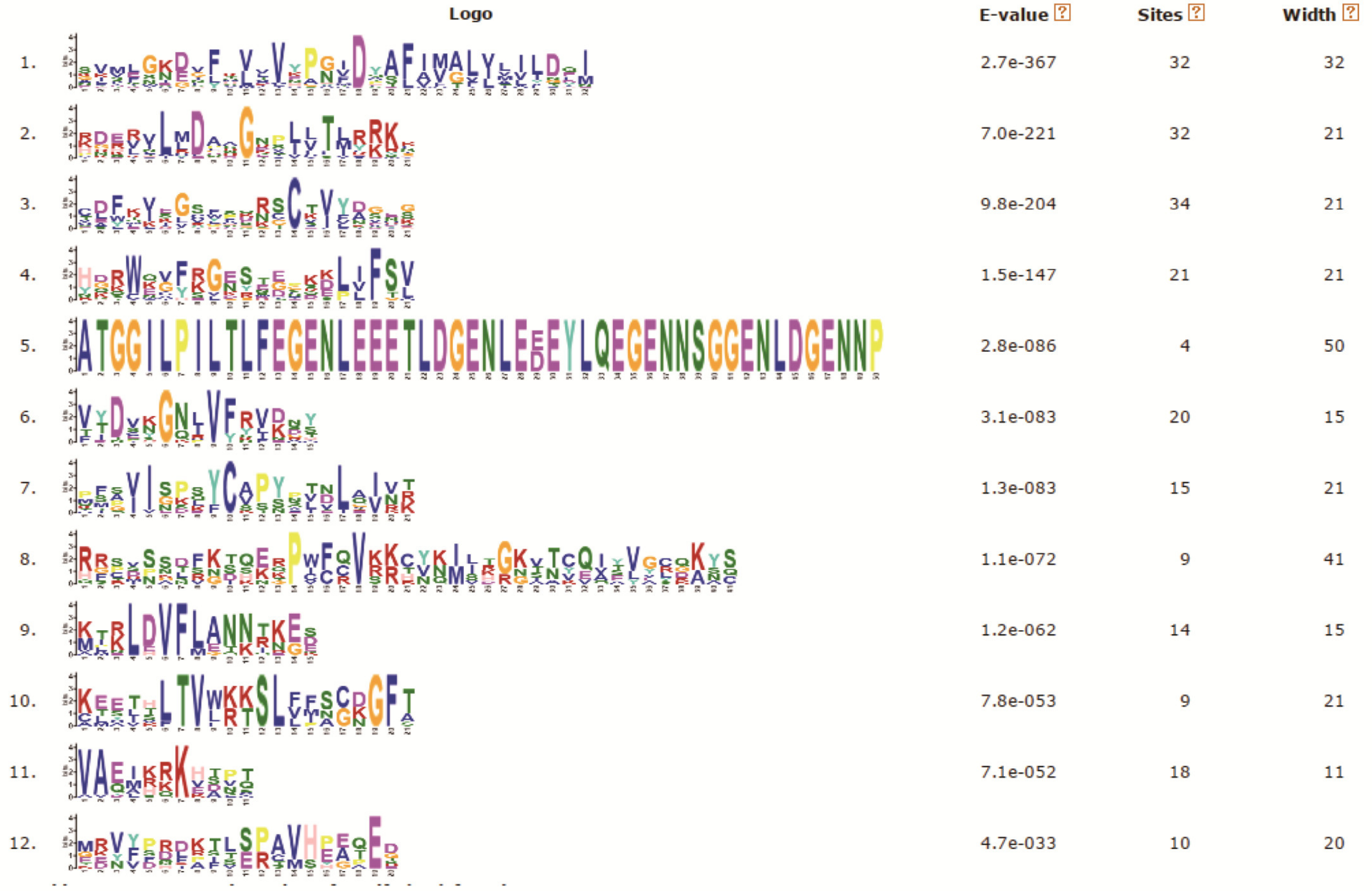

Figure S1. The motif logos of 12 motifs among GmLOR family proteins.

Table S1. Description of soybean LOR genes and their encoded proteins.

Number	Gene name	Photozome Soybean Gene ID	Genome Location	+/- strand	the number of intron	Transcripts number	Protein physicochemical characteristics		
							Length(aa)	MW(kDa)	Theoretical point (pI)
1	*GmLOR1*	*Glyma.01g129000*	Chr01:44261208-44263378	+	2	1	198	22.6	8.71
2	*GmLOR2*	*Glyma.01g129100*	Chr01:44287515-44289783	+	2	1	198	22.4	8.72
3	*GmLOR3*	*Glyma.01g218200*	Chr01:54828960-54830170	-	2	1	194	21.9	9.26
4	*GmLOR4*	*Glyma.01g222600*	Chr01:55141598-55142782	-	1	1	227	25.8	9.64
5	*GmLOR5*	*Glyma.02g233000*	Chr02:42057884-42060881	+	2	3	223	25.4	9.30
6	*GmLOR6*	*Glyma.02g260000*	Chr02:44682536-44683450	+	1	1	109	12.4	5.50
7	*GmLOR7*	*Glyma.03g040900*	Chr03:5164999-5166689	-	2	1	198	22.4	8.89
8	*GmLOR8*	*Glyma.03g041000*	Chr03:5173710-5176917	-	2	1	201	22.8	9.15
9	*GmLOR9*	*Glyma.05g206200*	Chr05:38904753-38908922	-	1	1	196	21.9	9.11
10	*GmLOR10*	*Glyma.05g212300*	Chr05:39373867-39375471	-	1	1	229	26.3	9.33
11	*GmLOR11*	*Glyma.07g030300*	Chr07:2419300-2420526	+	1	1	219	24.8	6.24
12	*GmLOR12*	*Glyma.07g045500*	Chr07:3790645-3792095	-	2	1	197	22.4	8.98
13	*GmLOR13*	*Glyma.08g013100*	Chr08:1027312-1028389	-	1	1	149	17.2	9.42
14	*GmLOR14*	*Glyma.08g018800*	Chr08:1515199-1517327	-	1	1	224	25.5	9.11
15	*GmLOR15*	*Glyma.08g212600*	Chr08:17146617-17147928	-	1	1	219	24.7	6.35
16	*GmLOR16*	*Glyma.09g070200*	Chr09:7157609-7159103	+	2	1	205	22.6	8.83
17	*GmLOR17*	*Glyma.09g278800*	Chr09:49414759-49415875	-	2	2	197	22.2	6.09
18	*GmLOR18*	*Glyma.09g279000*	Chr09:49431550-49433717	-	2	2	215	24.0	8.35
19	*GmLOR19*	*Glyma.11g020800*	Chr11:1480155-1481294	+	1	1	234	26.7	9.51
20	*GmLOR20*	*Glyma.11g025000*	Chr11:1767713-1768839	+	2	1	193	21.8	9.30

21	*GmLOR21*	*Glyma.13g111100*	Chr13:22468371-22469170	+	1	1	122	13.9	8.19
22	*GmLOR22*	*Glyma.13g365700*	Chr13:45171363-45172616	-	1	2	220	24.7	4.82
23	*GmLOR23*	*Glyma.14g054000*	Chr14:4275597-4277726	+	2	1	208	23.6	6.97
24	*GmLOR24*	*Glyma.14g200600*	Chr14:46555648-46558996	-	2	3	197	22.1	9.16
25	*GmLOR25*	*Glyma.15g007500*	Chr15:626771-628081	+	1	1	219	24.8	4.97
26	*GmLOR26*	*Glyma.15g178300*	Chr15:17001603-17004979	+	2	2	211	23.4	8.74
27	*GmLOR27*	*Glyma.16g013400*	Chr16:1163855-1165230	-	2	1	201	23.0	9.06
28	*GmLOR28*	*Glyma.16g069500*	Chr16:6948341-6950438	+	2	1	314	34.8	4.30
29	*GmLOR29*	*Glyma.16g069700*	Chr16:6957647-6959483	+	2	1	265	29.3	4.71
30	*GmLOR30*	*Glyma.16g070000*	Chr16:6977903-6981588	+	3	1	244	27.1	4.79
31	*GmLOR31*	*Glyma.16g070100*	Chr16:6989476-6991203	+	1	1	160	17.6	3.96
32	*GmLOR32*	*Glyma.16g222200*	Chr16:37858930-37859879	-	1	1	208	23.5	9.32
33	*GmLOR33*	*Glyma.17g048500*	Chr17:3672140-3674184	-	2	1	201	22.5	9.01
34	*GmLOR34*	*Glyma.18g209800*	Chr18:49526729-49530144	+	2	1	216	24.2	7.67
35	*GmLOR35*	*Glyma.18g209900*	Chr18:49531151-49532479	+	2	1	141	16.4	6.18
36	*GmLOR36*	*Glyma.18g210000*	Chr18:49538775-49540202	+	1	1	123	14.2	9.48
